# Gathering information on maternal immunization readiness in Bangladesh, Ghana, Kenya, Mozambique, and Nepal: Applying a WHO checklist

**DOI:** 10.1080/21645515.2024.2437258

**Published:** 2024-12-17

**Authors:** Ranju Baral, Sophia Knudson, Iracema Barros, Patience Cofie, Patience Dapaah, Sadaf Khan, Sandeep Kumar, Kamran Mehedi, Lauren Newhouse, Elkanah Otiang, Rosemond Owusu, Clint Pecenka, Melanie Picolo, Judite Pinto, Diana Quelhas, Satyabrata Routray, Surendra Uranw, Jessica A. Fleming

**Affiliations:** a Center for Vaccine Innovation and Access, PATH, Seattle, USA; b Primary Health Care, PATH, Seattle, USA; c Primary Health Care, PATH, Maputo, Mozambique; d Primary Health Care, PATH, Accra, Ghana; e Medical Devices and Health Technologies, PATH, Accra, Ghana; f India Country Program, PATH, New Delhi, India; g Center for Vaccine Innovation and Access, PATH, Dhaka, Bangladesh; h Epidemic Preparedness and Response, PATH, Nairobi, Kenya; i Independent Consultant, Accra, Ghana; j Independent Consultant, Maputo, Mozambique; k B.P. Koirala Institute of Health Sciences (BPKIHS), Dharan, Nepal

**Keywords:** Maternal vaccines, country readiness, checklist

## Abstract

New respiratory syncytial virus (RSV) maternal vaccines have begun roll out in some countries, with efforts in progress to broaden access worldwide and shorten the timeline to access for low- and middle-income countries (LMICs). Prior to new maternal immunization (MI) introductions, countries will need to evaluate their capacity and readiness for successful introduction. The World Health Organization’s Maternal Immunization and Antenatal Care Situation Analysis (MIACSA) project (2016–2019) developed a checklist for countries to self-evaluate their capacity to introduce new maternal vaccines. Here, we report on our use of the MIACSA checklist in Bangladesh, Ghana, Kenya, Mozambique, and Nepal to gather country stakeholders’ perceptions of MI readiness and provide additional considerations for implementers when using the checklist to optimize its usefulness.

## Background

Infectious diseases are a major cause of infant mortality worldwide and are responsible for 2.4 million neonatal and 2.6 million under five year deaths each year.^1,2^ While vaccination is an important tool for preventing many infectious diseases, this option typically provides insufficient protection against infectious diseases that have a high burden in the neonatal period.^3,4^ Vaccination during pregnancy, or maternal immunization (MI), is a promising way to protect pregnant populations, their infants, or both from certain infectious diseases.^5^ Vaccines against tetanus, influenza, and pertussis have long been used for MI in high-income settings; however, only tetanus toxoid-containing vaccines are widely available to immunize pregnant populations in low- and middle-income settings.

The first maternal vaccine against respiratory syncytial virus (RSV) – the world’s top cause of severe infant respiratory infection and hospitalization – is now licensed and rolling out in many upper- and some upper-middle income countries, with efforts in progress to broaden access worldwide and shorten the timeline to access for low- and middle-income countries (LMICs).^6–11^ Maternal vaccine candidates against other high-burden infectious pathogens for young infants are also in development, such as against Group B *Streptococcus* (GBS).^12–14^ With a growing portfolio of vaccines for use in pregnancy, assessing country readiness to introduce new maternal vaccines in LMICs is critical. MI has long been used as part of successful maternal and neonatal tetanus prevention programs across countries,^15^ laying the foundation for delivery of other vaccines in pregnancy. Low uptake of maternal vaccines outside of tetanus, however, highlights important challenges. While past and current MI delivery experiences (e.g., tetanus toxoid) can be leveraged, new maternal vaccines will have nuances that may require implementation adaptations – considering the natural intersection between immunization and maternal, newborns, and child health (MNCH) programs (i.e., antenatal care [ANC]). To access hard to reach pregnant populations, the Maternal and Neonatal Tetanus Elimination Initiative augmented routine immunization with supplementary immunization activities^15^ while new maternal vaccines are expected to be delivered primarily through routine systems. Also, while tetanus-diphtheria vaccine can be given at any time during pregnancy, the administration of RSV maternal vaccine is recommended within specific gestational age windows to optimize protection for the infant after birth.^8,10,16^ Timely delivery will require close coordination between the immunization and ANC programs to ensure pregnant women reach the health system during the recommended gestation period for vaccination.

In preparation for new maternal vaccines, the World Health Organization (WHO) initiated the Maternal Immunization and Antenatal Care Situation Analysis (MIACSA) study in 2016 to evaluate the maternal tetanus immunization program in LMICs and inform future MI introduction.^17^ The MIACSA project developed a”typology of health systems delivering vaccines to pregnant women, explored the attributes of successful programs currently vaccinating pregnant women, and assessed a path forward for introducing additional vaccines for pregnant women.”^17^ The study developed a checklist to support countries in self-evaluating their capacity and readiness to introduce maternal vaccines by identifying strengths and gaps in healthcare infrastructure as well as immunization and ANC programs. The checklist is also useful for identifying priority actions for successful routine use^18,19^ and can be used iteratively for monitoring progress against gaps over time. It is one tool among others that can contribute to MI introduction readiness assessments.^20,21^

We report here on our use of the MIACSA checklist in Bangladesh, Ghana, Kenya, Mozambique, and Nepal to gather country stakeholders’ perceptions of MI readiness and provide additional considerations for implementers to optimize its usefulness. We completed the MIACSA checklist with information collected from national stakeholder workshops conducted as part of MI cost of delivery (COD) studies in each country.

## Materials and methods

The MIACSA checklist includes 29 questions to evaluate country capacity and readiness to introduce a new maternal vaccine.^18^ Thematic areas covered by the checklist include policy and governance; immunization, ANC services, and coordination; vaccine delivery; disease surveillance; monitoring and evaluation; vaccine hesitancy; and demand creation. Each question is accompanied by rationale of how the question informs the assessment of country readiness. Sixteen of the 29 questions also include definitions of indicators. Implementers can rate each question as “yes,” “no,’ or “yes but needs strengthening prior to introduction.” We filled out the MIACSA checklist for each country based on inputs from country experts and program/policymakers following country-specific stakeholder workshops conducted as part of MI (COD) studies. Between 2022 and 2024, PATH, a global health nonprofit, initiated prospective COD studies for new maternal vaccines in Bangladesh, Ghana, Kenya, Mozambique, and Nepal. Countries were chosen to represent diversity in geography, infant RSV and GBS disease burden, and ANC and immunization system performance as measured by coverage of four or more ANC visits (ANC4+) by pregnant women and three doses of diphtheria, pertussis, tetanus vaccine (DPT3) in children, among others.^22,23^ To introduce the COD study, PATH organized country stakeholder workshops, which were co-hosted by respective countries’ Ministries of Health (MOHs); the Kenya Medical Research Institute (KEMRI) and B.P. Koirala Institute of Health Sciences also contributed in Kenya and Nepal, respectively. Workshop participants were purposively selected based on their professional positions and involvement with immunization or maternal health programs. They included representatives of the MOH; regional, provincial, and district health offices; immunization advisory bodies, including national immunization technical advisory groups (NITAGs); academics and professional associations; research organizations; and non-governmental organizations working in public health.

We completed checklists for each country based on interpretation of feedback from stakeholders participating in small thematic groups and plenary workshop discussions; individual stakeholders were consulted for clarification, as needed. A review of secondary data (i.e., Demographic and Health Surveys [DHS], Health Management Information Systems [HMIS], and other household surveys) and a literature review of relevant policies were also used for some quantitative indicators. The completed checklists were reviewed with respective country MOH representatives to confirm data validity and accuracy. Learnings from the stakeholder workshops are summarized in a separate paper.^24^

Here, we present completed MIACSA checklists from the five study countries, highlighting some themes that arose across the countries, our experience using the checklist, and considerations for countries using the checklist in the future. We indicate with an asterisk (*) when one country’s indicator is different than a common ranking in the other four countries and provide context in an added column titled “Further information.” This assessment is not designed or intended to provide cross-country comparison; rather to illuminate key areas for future consideration when evaluating readiness for new maternal vaccine introduction in different LMIC contexts.

## Results

Between 36 and 57 stakeholders participated in each country workshop, representing a variety of professions and stakeholders across immunization and maternal child health domains (Table 1). Completed MIACSA checklists for each country are presented in Table 2. Key findings in each thematic group are described below. The data filled in the checklist (Table 2) will continue to evolve as they are based on current knowledge and perspectives/subjective assessment of select representatives at the time of analysis.

**Table 1.** Number of participants in the maternal immunization cost of delivery study stakeholder workshop discussions by country that helped inform the MIACSA checklist completion exercise.

	Bangladesh	Ghana	Kenya	Mozambique	Nepal
Ministry of Health and regional/provincial/district health office representatives	25	25	19	18	19
Academic/research Organization, National Immunization Technical Advisory Group (NITAG) members, and professional association representatives	22	8	17	10	11
Non-Governmental Organization representatives	10	15	8	8	7
Participants (#)	**57**	**48**	**44**	**36**	**37**

**Table 2.** MIACSA checklist for assessing country readiness for maternal immunization programming based on workshop findings.

Indicator/Question	Bangladesh	Ghana	Kenya	Mozambique	Nepal	*Further information
**Policy**						
Is there an existing national policy on maternal tetanus immunization?	Yes, but needs strengthening	Yes	Yes	Yes, but needs strengthening	Yes	
Is there a national policy on number and content of antenatal care (ANC) visits?	Yes	Yes	Yes	Yes	Yes	
**Governance**						
Is there a functioning National Immunization Technical Advisory Group (NITAG)?	Yes	Yes	Yes	Yes	Yes	
If there is a NITAG, is there a Maternal Neonatal Child Health (MNCH) expert on the NITAG?	Yes	Yes	Yes	Yes	Yes, but needs strengthening*	MNCH experts are not regular members on the NITAG; however, they are invited for decisions on providing immunization to pregnant women.
If there is no MNCH expert on the NITAG is there engagement with the relevant expert group(s)?	Yes	Yes	Yes	Yes	Yes	
**Recent experience introducing additional maternal vaccines**						
Do you give any maternal vaccines in addition to Tetanus-Toxoid/Tetanus-diphtheria (e.g., Influenza, Pertussis, Yellow Fever, Meningococcal A)?	No	No	No	No	No	
**Recent experience introducing new vaccines**						
Have you introduced any new vaccine(s) in the National Immunization Program (NIP) in the previous 5 years?	Yes	Yes	Yes	Yes	Yes	
**Financing**						
Is the NIP able to mobilize and use sufficient resources for the existing program and its expansion without threatening financial sustainability of domestic funding?	No	No	No	No	Yes, but needs strengthening*	Upon validation of these findings, the MOH in Nepal noted that there are never enough resources, though they have been able to mobilize and expand the NIP in the past.
**Existing Tetanus Program**						
Does tetanus-toxoid 2+ doses (TT2+) coverage meet targets defined in the National Policy or World Health Organization (WHO) recommended coverage targets?	Yes*	No	No	No	No	According to the Coverage Evaluation Survey (CES) 2019, Bangladesh has a TT2+ coverage of 94.16%.^25^ WHO/UNICEF 2021 data reports TT2+ coverage in Bangladesh as 94%.^26^ Bangladesh is the only of the 5 countries meeting or surpassing the WHO target of 80%.^27,28^
Is the Protection at Birth (PAB) >90%?	Yes	No	No	No	Yes	
**Existing Immunization Program**						
Does Diphtheria Tetanus-Toxoid and Pertussis 3-dose (DTP3) coverage meet targets (per age group) defined in the National Immunization Policy?	Yes	No	Yes	No	No	Global immunization agenda 2030 target is to achieve 90% coverage of DTP3. National DTP3 coverage targets for each country are as follows: Bangladesh: 95% Ghana: 90%^29^ Kenya: 90% by 2026^30^ Mozambique: 90%
**Existing Antenatal Care**						
Does ANC 4+ visit (ANC4+) coverage meet targets as defined in the national antenatal care policy or internationally recommended coverage targets?	No	No	No	No	No	
Is a system in place to record the proportion of women who have at least one ANC visit in the 3rd trimester?	No*	Yes, but needs strengthening	Yes	Yes, but needs strengthening	Yes, but needs strengthening	The data can be retrieved manually but the information system does not organize it in a meaningful manner.
Is a system in place to record the proportion of women who have at least one ANC visit in the 2nd trimester?	No*	Yes, but needs strengthening	Yes	Yes, but needs strengthening	Yes, but needs strengthening	The data can be retrieved manually but the information system does not organize it in a meaningful manner.
Is a system in place to record ANC quality?	Yes	No	No	Yes	No	
Are there any ANC fees currently incurred by pregnant women?	No	Yes, but needs strengthening*	No	No	No	The ideal answer to this question would be No, indicating that all ANC visits are free to pregnant women. Ghana, however, reported “Yes, but needs strengthening” because 4 ANC visits are free per their national policy, while a pregnant woman may incur costs if she is ill or needs laboratory testing or treatment. Since some instances exist where pregnant women pay for indirect costs during ANC visits, Ghana reported that they would like to strengthen the system to eliminate these fees in the future.
**Program coordination**						
Are program coordination, roles and responsibilities by NIP and ANC/MNCH clearly defined and understood?	Yes, but needs strengthening	Yes, but needs strengthening	Yes, but needs strengthening	Yes, but needs strengthening	Yes, but needs strengthening	
**Delivery Platform**						
Is a program in place for health worker (HW) education and training in relation to maternal immunization?	Yes, but needs strengthening	Yes, but needs strengthening	Yes*	Yes, but needs strengthening	Yes, but needs strengthening	Organized continuing medical education and mentorship programs for health workers on all immunization programs are in place in Kenya.
Is there an adequate number of HWs to deliver new maternal vaccines without compromising delivery of existing components of antenatal care?	Yes	Yes	No	Yes	Yes, but needs strengthening	
**Vaccine delivery system**						
Is the vaccine procurement system robust and able to forecast demand for all existing and new vaccines?	Yes	Yes	Yes	Yes	Yes	
Is the cold chain capacity adequate for existing vaccines and for the addition of a new vaccine?	Yes*	Yes, but needs strengthening	Yes, but needs strengthening	Yes, but needs strengthening	Yes, but needs strengthening	No reason given.
**Surveillance**						
Is surveillance for maternal mortality and Vaccine Preventable Diseases (VPDs) in place?	Yes, but needs strengthening	Yes, but needs strengthening	Yes	Yes	Yes, but needs strengthening	
Is surveillance for neonatal mortality and VPDs in place?	Yes, but needs strengthening	Yes, but needs strengthening	Yes	Yes	Yes, but needs strengthening	
Is stillbirth surveillance in place?	Yes	Yes, but needs strengthening	Yes, but needs strengthening	Yes	Yes, but needs strengthening	
Is an Adverse Effects Following Immunization (AEFI) reporting system in place?	Yes, but needs strengthening	Yes, but needs strengthening	Yes	Yes	Yes, but needs strengthening	
Does the AEFI reporting have the capacity to identify pregnant women?	No	Yes, but needs strengthening	No	Yes	Yes	
**Monitoring and evaluation**						
Is a health information system (including maternal vaccination) in place? If there is a health information system in place, does it capture maternal vaccination by private providers?	Yes	Yes, but needs strengthening*	Yes	Yes	Yes	The health information system is in place in Ghana, but it does not currently capture private providers.
**Vaccine hesitancy**						
Is there evidence of vaccine hesitancy among your pregnant population?	No	Yes	Yes	No	No	
**Demand creation/communication**						
Are demand creation activities for maternal immunization such as a communication strategy planned and/or in place?	No	Yes, but needs strengthening	No	No	Yes, but needs strengthening	

Note: The data filled in this table will continue to evolve as they are based on current knowledge and perspectives/subjective assessment of select representatives at the time of analysis.

*Indicates an instance where one country’s response differed from the other four countries. These instances are explained in further detail in the last column of this table and in the results section.

### Policy, governance, recent vaccine introduction and financing

Stakeholders in all five countries reported having a national policy or strategic plan on maternal tetanus immunization and governance structures that support MI (Table 2). Similarly, all countries reported having a policy on the number and content of ANC visits that could be adapted to new interventions. All countries have active and functioning national immunization technical advisory groups (NITAGs) and stakeholders reported experts in MNCH being involved in NITAG meetings when needed.

Regarding immunization financing, stakeholders in four countries reported that their national immunization programs (NIPs) have insufficient resources for existing services and voiced concern that the addition of new vaccines might threaten the financial sustainability of the program. Nepal was the only country to report having sufficient resources but needing strengthening prior to introduction, based on previous success in mobilizing resources for the immunization program, though noting that there are never enough resources. Although all countries are currently eligible for immunization funding through Gavi, the Vaccine Alliance, Bangladesh, Ghana, and Kenya are in the final, or accelerated, transition phase to self-financing (Table 3). The increase in the share of country co-financing to supplement Gavi resources for immunization program was identified as one of the major challenges related to immunization financing and sustainability, particularly for countries in the accelerated Gavi transition phase.

**Table 3.** Countries’ transition status from Gavi, the vaccine alliance support as of 2022*.

Country	Funding Phase	Definition of funding phase
Bangladesh	Accelerated transition (Phase 3)	Countries within gross national income per capita above the US$1,730 threshold when its three-year average gross national income as well as its most recent gross national income are above the eligibility threshold, and the country is co-financing at least 35% of vaccine costs
Ghana		
Kenya		
Mozambique	Initial self-financing (Phase 1)	Countries within gross national income per capital equal to or below US$1,085
Nepal	Preparatory transition (Phase 2)	Countries within gross national income per capita between US$1,085 and US$1,730

*Information collected from Gavi, The Vaccine Alliance Vaccine Application Process Guidelines (2023).^38^

### Existing immunization and antenatal care programs

Protection at birth (PAB) and tetanus-toxoid 2+ doses (TT2+) coverage are indicators of the existing maternal tetanus program performance whereas Diphtheria Tetanus-Toxoid and Pertussis 3-dose (DTP3) coverage indicated childhood immunization performance. Based on WHO and DHS data, Bangladesh and Nepal were the only countries to meet the target for PAB for tetanus, at or above 90%.^26^ According to WHO data, Ghana met the 90% PAB target according to WHO data but not when using the DHS data for 2022 (74%).^26,31^ Target TT2+ coverage of 80% was only met in Bangladesh (94%).^25–28^ Bangladesh and Kenya met the national DTP3 coverage targets (≥90%).^30,32^ Bangladesh met all national or global immunization coverage targets on the MIACSA checklist.^25,26,32^

The MIACSA checklist includes coverage of four or more ANC visits (ANC4+) as an indicator of ANC program strength, rather than the updated eight visits currently recommended by WHO.^23^ All countries fell below the global target of 80% ANC4+ visits.^31,^^33–37^

Participants from Bangladesh and Mozambique reported having a system in place to record ANC quality, despite the checklist acknowledging the lack of a commonly used definition of ANC quality.^18^ Most countries reported having a system that records gestational age at the time of the initial ANC visit, though most countries do not analyze this data to understand ANC visits by trimester or gestational age. Kenya was the only country that reports on ANC visits by gestational age due to facility ANC data integration with the Kenya Health management Information System (KHMIS).

### Program coordination, delivery platform, and vaccine delivery system

All five countries mentioned no clear delineation of the responsibilities of MI program coordination between the National Vaccines and Immunizations Program (NVIP) and ANC/MNCH programs. The pre-service curricula for health workers providing both immunization and ANC services usually include both components enabling inter-changeability and transition between the roles. All countries reported such efforts as ‘needing strengthening,’ acknowledging the importance of having clear roles, responsibilities, and communication pathways between NVIP and ANC to ensure functional integration of MI into the broader immunization program.

Given the flexibility of health workers across programs, most countries self-assessed as having enough health worker positions to support the delivery of new maternal vaccines. However, these stakeholders acknowledged existing challenges that may affect staffing availability. Stakeholders from Nepal highlighted the expectation for MI to be performed by immunization program staff, but shared concerns about inadequate human resourcing due to a shortage of health workers and heavy workloads with existing health workers and ANC staff. Similarly, Bangladesh reported a large share of vacant health worker positions. Mozambique shared the availability of maternal health nurses in each health facility, and their experience with COVID-19 vaccination having adequate staff support, however stakeholders felt unsure if the current staffing is adequate for MI. Stakeholders from Ghana did not share these concerns, and instead emphasized that confidence would be higher among community health workers that routinely perform vaccination. Kenya was the only country that reported an inadequate number of health workers to deliver new maternal vaccines due to a lack of financial resources that have limited continuous recruitment of new health workers.

Bangladesh stakeholders reported adequate cold chain capacity for existing and new vaccines; conversely, Kenyan stakeholders indicated insufficient freezer space and the need for additional equipment across the country. Participants in Nepal reported inadequate storage at the central store, but sufficient capacity at the primary health care level. Stakeholders in all countries reported substantial improvements in cold chain capacity resulting from the COVID-19 pandemic.

### Surveillance, monitoring, and evaluation

Stakeholders from all countries reported having surveillance systems that monitor maternal and neonatal mortality, vaccine-preventable diseases, stillbirths, and adverse events following immunizations (AEFIs), though three countries reported these needing strengthening. Adding additional facilities contributing data to surveillance was a common suggestion across country stakeholders as a means of strengthening their systems. Despite having a system for reporting AEFIs in all countries, only stakeholders from Mozambique and Nepal reported the ability to identify pregnant women in their systems.

### Vaccine hesitancy, demand creation, and communication

Stakeholders in Ghana and Kenya reported vaccine hesitancy increasing over the COVID-19 pandemic, primarily among pregnant women and religious groups. Stakeholders in Bangladesh, Mozambique, Nepal, however, reported no or minimal hesitancy in their populations.

Stakeholders in Bangladesh, Kenya, and Mozambique were not aware of any direct demand-creation activities or communication strategies in place to support maternal vaccine uptake. Stakeholders from Ghana and Nepal noted specific activities with the aim of improving MI coverage. Stakeholders from Ghana mentioned Social Behavioral Change Communications campaigns that specifically included MI within their activities, though noted that the focus was minimal.

## Discussion/conclusions

The MIACSA checklist is a tool to assess strengths and areas needing improvement of a country’s health system relevant to the introduction of a new maternal vaccine and to help identify priority actions to achieve readiness.^18^ The checklist supports national decision-making and planning efforts around the introduction of new maternal vaccines and can be used to track progress on preparation over time. We opportunistically used stakeholder workshops conducted as part of a multi-country MI COD study to complete MIACSA checklists for five study countries, providing a snapshot of the status of MI introduction readiness across these countries based on our interpretation of feedback from workshop stakeholders. To our knowledge, this is the first application of the checklist in countries beyond piloting during the initial MIACSA project.^18^

### Strengths and opportunities for MI

The country checklists highlight several strengths and opportunities within the existing health systems in preparation for MI introductions. All countries responded positively to having a national policy on MI, a functioning NITAG with MNCH expertise when needed, successful introduction of at least one new vaccine in recent years, and a robust vaccine procurement system, all of which provide a strong foundation to integrate new maternal vaccines within the existing system. We found opportunities to strengthen health systems through improving program coordination (e.g., developing clear roles and responsibilities for NIP and MNCH staff), increasing cold chain capacity, expanding surveillance for vaccine preventable diseases, and strengthening monitoring and evaluation.

### Areas requiring further strengthening

We identified several common areas most in need of improvement, indicated by three or more countries reporting “No” to a checklist question. First, more than half of the countries are not currently meeting national and/or WHO recommended coverage targets for existing maternal TT2+, PAB, DPT3, and ANC4+ coverage – underscoring a need to strengthen current programs to enable successful MI introduction. Second, most immunization programs do not have sufficient resources for existing vaccines and would struggle to add new vaccines without threatening the stability of domestic funding. Having sustainability plans for immunization funding beyond Gavi support is, therefore, critical. Lastly, recording systems to capture ANC quality and demand creation activities for MI are lacking in most settings. Strengthening recording systems and establishing communications strategies to build trust and understanding in the community about the needs and objectives of an MI program may also be needed.

Additional areas of improvement were identified by at least three countries reporting “No” or “Yes, but needs strengthening” to the corresponding checklist question. These include cold chain; health worker hiring and training on MI; delineation of responsibilities between EPI and MNCH programs for MI; MNCH expert representation within routine NITAG meetings; updating health information systems to routinely record and report the proportion of women attending ANC visits by trimester or gestational age; and updating vaccine safety monitoring systems to capture maternal mortality, neonatal mortality, stillbirth, and AEFIs generally and among pregnant women.

### Observations from using the MIACSA checklist

Our experience using the MIACSA checklist may be useful to others. We found the checklist easy to use and comprehensive. As a standardized document, it can be used across countries and vaccines. Each question provides a rationale for its importance, which is helpful as countries contextualize MI readiness.

While many of the questions in the checklist have helpful definitions, nearly half do not include precise definitions of indicators, requiring countries to create their own definitions and leaving room for differences in interpretation. Aligning on understanding of each question and agreeing upon thresholds for ranking “Yes,” “No,” and “Yes, but needs strengthening” prior to completing the tool may be useful to future users.

While the checklist’s content is comprehensive for evaluating overall MI readiness, its ability to capture within-country nuances and variations is limited. The answer choices in the checklist is limited to “Yes,” “No,” and “Yes, but needs strengthening.” To capture the nuances and complexities, countries could consider adding follow-up, open-ended questions and/or a notes column to document context-specific information, relevant findings, actions/solutions, or guide prioritization of next steps.

## Conclusions

The MIACSA checklist was intended for countries to self-assess their system readiness for introducing new maternal vaccines, designed to be standardized and agnostic to country or vaccine, allowing generalizability. We expanded this use case by applying it to identify common strengths and gaps across multiple countries. Differences in interpretation and understanding of questions and answers, however, limited our ability to use the checklist as a cross-country comparison tool.

MIACSA guidance around identifying appropriate individuals to complete the checklist includes ‘national stakeholders’ as well as a local pilot team, including members from the MOH, NIPs, and MNCH programs.^18^ We found that involving the appropriate stakeholders and providing a forum for contextualizing questions and answers while encouraging open discussion was important to build consensus within stakeholder groups. Perspectives from program implementers at the peripheral health facilities is critical to get deeper insights on implementation issues and accurate assessment of readiness. There may be additional value for individuals participating in the completion of the checklist to assign themselves to be responsible for identified gaps and action items to add accountability to proposed next steps.

We opportunistically used stakeholder convenings from a costing study to engage ministries of health and stakeholders to complete the MIACSA checklist on MI country readiness, identifying both common and context-specific areas for strengthening across various LMIC contexts. We found the MIACSA checklist to be a useful tool to help countries establish a baseline and periodically evaluate MI introduction preparedness. Its use would benefit from early definition of stakeholders, audiences, and terminology to ensure consistency and comparability of findings over time within a country. The checklist should be used in complement with other country readiness assessment efforts to ensure a holistic picture of what will be needed to ensure successful introduction of new maternal vaccines. The ultimate decisions to introduce will require additional prioritization among other competing public health priorities and additional resources for implementation.

## Bioethical considerations

The study protocol for hosting the stakeholder workshop and the costing study were approved by respective ethics review committees in each study country: Bangladesh Medical Research Committee (Bangladesh); Ghana Health Service Ethics Review Committee (Ghana); Kenyatta National Hospital-University of Nairobi Ethics Review Committee (Kenya); National Bioethical Committee (Mozambique); and Nepal Health Research Council and BP Koirala Institute of Health Sciences Ethics Review Committees (Nepal).
